# Vaginal microbiota and gynecological cancers: a complex and evolving relationship

**DOI:** 10.1186/s13027-024-00590-7

**Published:** 2024-06-14

**Authors:** Kasra Javadi, Elaheh Ferdosi-Shahandashti, Mehdi Rajabnia, Mansoor Khaledi

**Affiliations:** 1 Department of Microbiology, Faculty of Medicine, Shahed University, Tehran, Iran; 2 Biomedical and Microbial Advanced Technologies Research Center, Health Research Institute, Babol University of Medical Sciences, Babol, Iran; 3 Infectious Diseases and Tropical Medicine Research Center, Health Research Institute, Babol University of Medical Sciences, Babol, Iran; 4 Department of Microbiology and Immunology, School of Medicine, Shahrekord University of Medical Sciences, Shahrekord, Iran

**Keywords:** Microbiota, Microbiome, Gynecological cancers, Cervical cancer, Ovarian cancer, Endometrial cancer, Vaginal cancer, Vulvar cancer

## Abstract

The vagina hosts a community of microorganisms known as the vaginal microbiota. This community is relatively stable and straightforward, with *Lactobacillus* species being the most dominant members. The vaginal microbiota has various functions that are essential for maintaining human health and balance. For example, it can metabolise dietary nutrients, produce growth factors, communicate with other bacteria, modulate the immune system, and prevent the invasion of harmful pathogens. When the vaginal microbiota is disrupted, it can lead to diseases and infections. The observed disturbance is distinguished by a reduction in the prevalence of *Lactobacillus* and a concurrent rise in the number of other bacterial species that exhibit a higher tolerance to low oxygen levels. Gynecologic cancers are a group of cancers that affect the female reproductive organs and tissues, such as the ovaries, uterus, cervix, vagina, vulva, and endometrium. These cancers are a major global health problem for women. Understanding the complex interactions between the host and the vaginal microorganisms may provide new insights into the prevention and treatment of gynecologic cancers. This could improve the quality of life and health outcomes for women.

## Introduction—what is the scope of this review?

The word “microbiota” was first used in the early 1900s, when various microorganisms, such as bacteria, viruses, and yeasts, lived in different parts of the human body [1]. Despite their frequent interchangeability, microbiota and microbiome have subtle differences in meaning. For instance, “microbiota” refers to microorganisms living in a particular habitat. On the other hand, the term “microbiome” refers to a more comprehensive notion that comprises not only the actual microbes but also their genomes, or genetic material, and the environments in which they live [2]. The microbiota is critical in maintaining human health and balance through many processes. These include several processes, such as acquiring energy from nutritional sources, promoting growth hormone synthesis, intercellular communication among bacteria, augmenting immune system activity, inhibiting pathogen colonisation and resisting their displacement of the epithelium or mucosal peithelium [3, 4].

The vaginal microbiota refers to a complex assemblage of microorganisms that inhabit the vaginal environment. Compared to other parts of the body, the vaginal microbiota is comparatively less varied and is often dominated by the *Lactobacillus* species [5, 6]. The probiotic activity is attributed to individual *Lactobacillus* species and their multi-microbial interaction [7]. Through competition with other bacteria for resources and the release of chemicals that make the vaginal environment inhospitable to other potentially harmful germs, the vaginal microbiota plays a crucial role in the health condition of the female reproductive system [8, 9]. Vaginal microbial dysbiosis contributes to the pathogenic process of illnesses. Frequently, there is an observed transition from a state characterised by a predominance of *Lactobacillus* to one marked by an elevation in facultative and anaerobic microorganisms [10, 11]. Treatments aimed at altering vaginal microbiota have been very successful in curing a variety of illnesses, which supports the viability of using vaginal microbiota in the management of gynecologic disorders [12].

Gynecologic cancers are a significant global health issue for women. The phrase “gynecological cancers” encompasses a range of malignant diseases that primarily affect the reproductive tissues and organs in females, such as the ovaries, uterus, cervix, vagina, vulva, and endometrium. Frequently, there is a transition from a state characterised by a predominance of *Lactobacillus* to one marked by an escalation in facultative and anaerobic microorganisms [13]. Gynecologic malignancies, which comprise about 14.4% of newly diagnosed cancer cases in women globally, have a substantial impact on both mortality and morbidity. The elements mentioned significantly influence the quality of life (QoL) experienced by survivors [14]. Patients have a diverse array of symptoms, including psychological anguish, menopausal complications, exhaustion, sleep disturbances, bladder and bowel dysfunction, lymphedema, and sexual impairments [15].

## Vaginal microbiota

The identification of vaginal microbiota dates back to 1892, when Albert Doderlein, a German obstetrician and gynecologist, observed the presence of elongated, robust, mobile, rod-shaped bacteria in vaginal fluid. These bacteria were characterised as gram-positive and non-spore-producing [16]. After discovering its ability to produce lactic acid, Doderlein’s bacillus was called *Lactobacillus*. Doderlein also observed that puerperal fever was connected to the lack of his bacillus in vaginal fluid and that it inhibited Staphylococcus growth [17, 18]. The findings of this study have shown that *Lactobacillus* is the predominant bacterial species in the vaginal microbiota and has a crucial role in maintaining women’s health [19].

The composition of the bacterial community in the vagina has been comprehensively determined to mainly consist of lactobacilli, which may be categorised into five unique groups referred to as community state types (CST). The dominant species in each of the classed vaginal microbiota states, known as CSTs I, II, III, IV, and V, are *Lactobacillus crispatus*, *L*. *gasseri*, *L*. *iners*, a polymicrobial flora consisting of *Lactobacillus* and bacteria associated with bacterial vaginosis (BVAB), and *L*. *jensenii*, respectively [19, 20]. Females usually exhibit CST I, III, and IV, which have been extensively investigated in scholarly studies. Conversely, CST II and V are less commonly seen in women [21]. Indeed, studies conducted by DiGiulo et al. and van de Wijgert et al. have shown that the vaginal microbiota of women in good health may be partly categorised into CST II and V [22, 23]. Gajer et al. (2012) further categorised CST IV, a notable deficiency in a specific *Lactobacillus* species, into two subgroups, CST IV-A and CST IV-B. According to the findings of Gajer et al., it has been shown that CST IV-B has a much higher abundance of BVAB. In contrast, CST IV-A generally consists of a modest proportion of *L*. *iners* and anaerobic bacteria such as *Corynebacterium*, *Finegoldia*, *Streptococcus*, or *Anaerococcus* [19, 24].

CST I is often linked to the preservation of vaginal health, which is crucial for preventing infections such as bacterial vaginosis, as well as other health concerns such as female infertility, early birth, and miscarriage. The main contributing factor to this phenomenon is its ability to produce lactic acid, hydrogen peroxide, and bacteriocins, creating an inhospitable environment for certain pathogenic microorganisms [25–27]. CST II, like CST I, generates lactic acid and plays a role in maintaining a favorable vaginal milieu. However, it serves a more dynamic and intermediate function in terms of protection [28]. The efficacy of CST III in protecting bacterial vaginosis and problems related to pregnancy is comparatively lower when compared to other species of *Lactobacillus*. There exists a correlation between the presence of *L*. *iners* and a vaginal environment that is susceptible to dysbiosis [29]. According to Witkin et al., many factors are believed to contribute to this phenomenon. One such factor is the ability of *L*. *iners* to produce a distinct isomeric form of lactic acid (L-lactic acid) that lacks the required power to hinder the invasion of pathogenic bacteria [30]. The absence of D-lactic acid seems to have a role in the deterioration of the extracellular matrix, hence facilitating the mobility of pathogenic microbes [31]. Moreover, it was also shown that *L*. *iners* generates inerolysin, a pore-forming protein often seen in disease-causing bacteria, which might increase its adhesive ability [32]. The ability of *L*. *iners* to generate inerolysin might be one of the most significant determinants impacting its capability to get nutrients from the vaginal environment [33]. To thrive in the bacterial vaginosis environment, *L*. *iners* can enhance the expression of inerolysin and mucin and stimulate the creation of glycerol and the expression of relevant metabolic enzymes. This allows the bacteria to obtain nutrients from external sources effectively [34, 35]. CST IV is correlated with vaginal dysbiosis, perhaps rendering women more susceptible to recurring infections. This association may be attributed to the likely creation of biofilm as well as an elevated risk of contracting sexually transmitted diseases and bacterial vaginosis [36]. The cytotoxic protein vaginolysin, dependent on cholesterol, is secreted in the highest amounts by CST IV, particularly by *G*. *vaginalis* [37]. Another group of bacteria associated with bacterial vaginosis, Fusobacteria and *G*. *vaginalis*, secrete the enzyme sialidase. This enzyme causes mucus breakdown, making the cervical epithelium more susceptible to viral infections [38, 39]. Finally, CST V is seen as a vaginal community state type that promotes a favorable and stable vaginal environment, akin to CST I [40].

Research has shown that the incidence of CST varies based on an individual’s ethnic origin. CST IV was primarily identified in black and Hispanic women, but the other four groups (I, II, III, and V) were mainly separated from white and Asian women. Additionally, women’s vaginal pH varied by ethnic group, with Hispanic and black women having higher vaginal pHs than Asian and white women [20, 41].

Extensive interplay occurs between the host immune system and the microbiota. The recruitment of natural killer cells, macrophages, and T and B lymphocytes occurs as a result of microbial activation of toll-like receptors located on the vaginal epithelial cells. The chemokines include interleukins such as IL-1β, IL-8, and IL-10, as well as tumor necrosis factor-a (TNF-α). The study revealed that women who exhibited a dominant CST IV had significantly higher levels of IL-1α, IL-1β, and IL-8 than those with dominant CST I communities. Additionally, women with dominant *L*. *iners* communities (CST III) had moderate amounts of IL-8 compared to those with CST I. Chemokines may exhibit variability due to transitions between cervical microbial community state types (CSTs). For instance, women transitioning from CST I to CST III and CST IV demonstrated increased levels of IL-1α, IL-1β, and TNF-α with time [42, 43]. Table 1 lists the various CST categories.

**Fig. 1:**
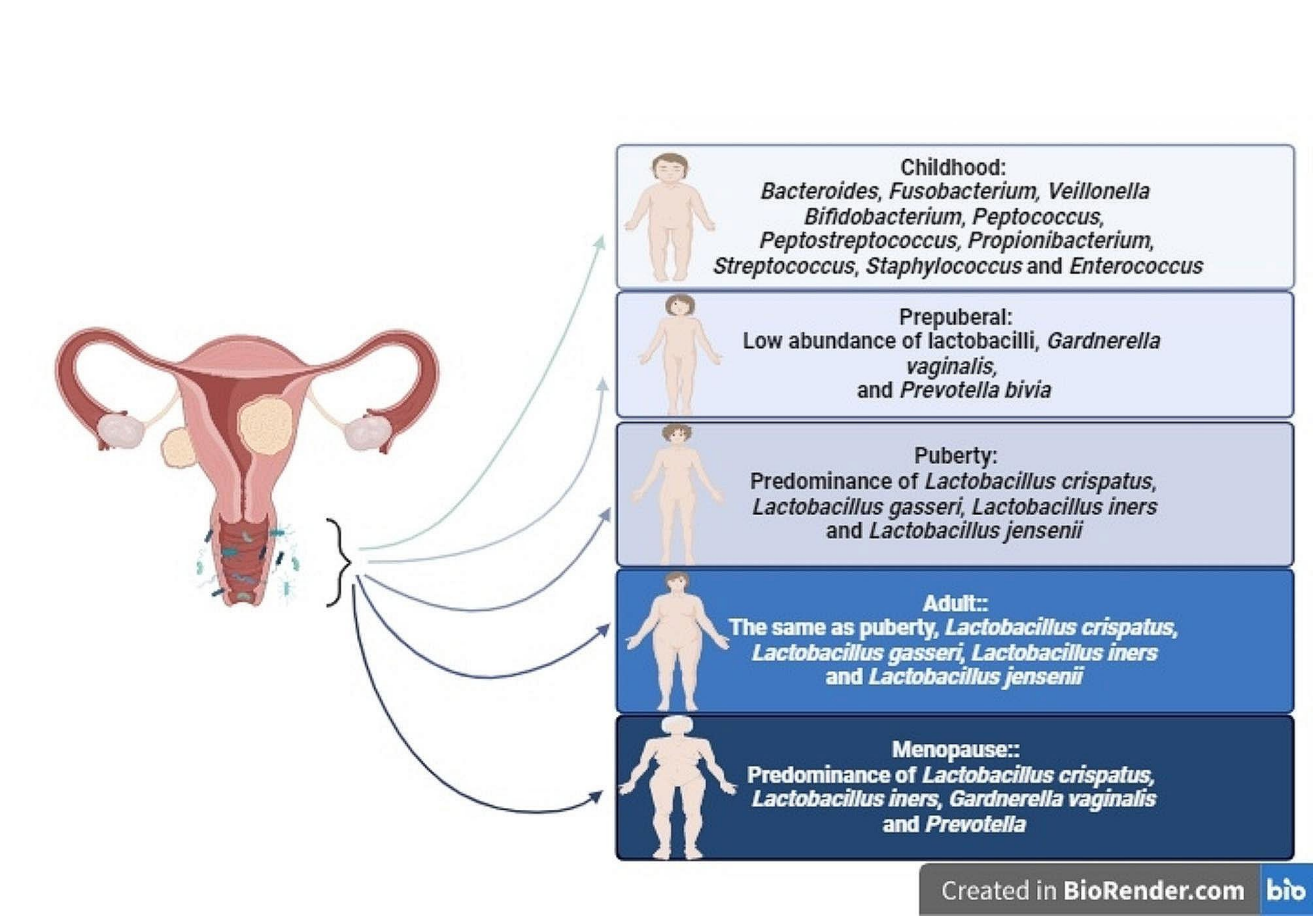
Alteration of the female vaginal microbiota throughout several life phases

The vaginal microbiome of women is a multifaceted and ever-changing micro-ecosystem that transforms their lifespan and menstrual cycle. The vaginal mucosa is stratified into a squamous, non-keratinized epithelium and cervicovaginal discharge [8, 44]. Researchers must examine the human microbiome to determine whether microbial populations are consistent or vary by individual to understand the human body. Human health and sickness risk may be linked to core microbiota alterations [45]. The female vaginal microbiota may alter throughout several life phases, such as childhood, prepuberal, puberty, adulthood and menopause. In actuality, the prevalent elements that guided the temporal variations in human vaginal microbiota were menstruation, vaginal douching, uncontrolled antibiotic use, and hormonal fluctuations [46]. In childhood, vaginal pH is neutral, and several bacteria species, including gram-negative anaerobic, gram-positive anaerobic, and aerobic bacteria, predominate in the vaginal environment. Following the period of childhood, the vaginal epithelial cells undergo stimulation by the hormone estrogen, resulting in the production of glycogen. This process ultimately leads to the establishment of a dominant population of lactobacilli in the healthy vagina throughout the reproductive years. In the menopause stage, there is a loss in estrogen levels, resulting in a subsequent reduction in glycogen synthesis. The hormonal fluctuation leads to a decrease in the population of lactobacilli. Nevertheless, even during menopause, *Lactobacillus* spp. persists as a prominent presence in the vaginal microbiome, although with increased diversity in comparison to earlier stages of life [47, 48] (Fig. 1).

Recent research has undertaken a comparative examination of the vaginal microbiota’s makeup at three unique locations inside the vagina: the cervix, mid-vagina, and introitus. These studies have shown that the female vaginal ecosystem consists of a wide range of more than 200 phylotypes, with the most common taxa belonging to the phyla Firmicutes, Bacteroidetes, Actinobacteria, and Fusobacteria [49].

**Table 1:** Community state type of the vaginal microbiota

CST	Dominate bacteria	Metabolic products	Ethnic origin	pH	Immune response
CST I	*L. crispatus*	Highest lactic acid, hydrogen peroxide, and bacteriocins	White and Asian women	4.0 ± 0.3	Not significantly raise pro-inflammatory cytokines
CST II	*L*. *gasseri*	Lowest lactic acid	Uniformly dispersed	5.0 ± 0.7	Induce low levels of some pro-inflammatory cytokines
CST III	*L*. *iners*	Moderate lactic acid and inerolysin	Asian women	4.4 ± 0.6	Induce moderate levels of IL 1α, IL-1β, TNF α, IL-8, and IL-10
CST IV	Non-*Lactobacillus*-dominated	Low lactic acid, vaginolysin and sialidase	Black and Hispanic women	5.3 ± 0.6	Induce higher levels of IL 1α, IL-1β, TNF α, IL-8, and IL-10
CST V	*L. jensenii*	Moderate lactic acid	White and Asian women	4.7 ± 0.4	Not significantly raise pro-inflammatory cytokines

## Vaginal microbiota in gynecological cancers

Gynecologic cancer encompasses malignancies that arise from the female reproductive organs. There are five main categories of gynecologic cancer, including cervical, ovarian, endometrial, vaginal, and vulvar cancer [50]. The primary occupants of the vaginal microbiome consist of organisms belonging to the Firmicutes phylum and *Lactobacillus* genus. Evidence suggests a correlation between the development of gynecological malignancies and alterations in the microbial community, namely a reduction in commensal bacteria and an elevation in anaerobic bacteria [51, 52].

### Cervical cancer

Cervical cancer, characterised by the malignant conversion of cells inside the cervix, which serves as the passage between the vagina and the uterus, has emerged as a notable public health issue in the contemporary era. The onset of this condition occurs when healthy cells undergo uncontrolled proliferation, resulting in the development of malignant tumors that can spread and cause harm to neighbouring vital organs [53]. In the year 2018, cervical cancer had a global impact on more than 500,000 women, leading to an estimated 300,000 fatalities. Cervical cancer ranks as the fourth most frequent kind of cancer among women [54]. According to projections made by the World Health Organization (WHO), it was estimated that 2020 there would be around 604,000 newly reported cases and 342,000 fatalities attributed to this particular ailment. If no significant intervention is implemented, it is projected that the worldwide prevalence of cervical cancer will increase to around 700,000 cases by the year 2030, resulting in an estimated 400,000 deaths. These findings indicate a rise of 21% in incidence rates and a 27% increase in fatality rates when compared to the data from 2018 [55].

The vaginal microbiota can influence the acquisition and long-term presence of the Human Papillomavirus (HPV), a viral infection that is linked to the development of cervical cancer [56]. HPV is generally recognized as the primary risk factor associated with the onset of cervical cancer since over 90% of cervical cancer cases are potentially linked to an HPV infection [57, 58]. HPV types 16 and 18 have been recognized as the most high-risk kinds and are linked to gynecologic malignancies and cervical cancer in women worldwide [59]. The known scientific literature has firmly proven the association between papillomaviruses and the transformative effects of the viral E6 and E7 oncoproteins [60, 61]. The protein encoded by the E6 oncogene can elicit cellular changes in the host cells. This phenomenon primarily targets the p53 protein, and its suppression may result in the development of malignant tumors and hinder the process of programmed cell death, known as apoptosis [62]. Conversely, the E7 protein has a complementary activity to the Retinoblastoma protein (PRb). The expression of E7 leads to the release of E2F, hence inducing DNA synthesis [63].

The cervical microenvironment is a multifaceted structure that includes immune cells and unique bacteria, which collaborate to orchestrate localized immune responses [64, 65]. Considerable research efforts have been dedicated to examining the intricate attributes of the cervical microenvironment to comprehend the interaction between the vaginal microbiota, HPV infection, various phases of cervical intraepithelial neoplasia (CIN), and cervical cancer. The research above indicates that an augmentation in bacterial variety and a reduction in lactobacilli may result in immunological dysregulation [66–68]. The phenomenon above gives rise to a milieu that is favorable for the proliferation of tumors, hence emphasizing the noteworthy influence of the vaginal microbiota as a contributing element in the progression of cervical cancer. The prevailing consensus in the scientific community is that while human HPV plays a significant role in the development of cervical cancer, it is not the only factor responsible for its occurrence [69].

Chen et al. studied Chinese cohorts to examine the connections between the vaginal microbiome, cervical cancer, CIN, and HPV infection. The study contrasted the makeup of vaginal microorganisms. Regardless of CINs, it was discovered that HPV infection enhanced the variety of vaginal microorganisms. Vaginal bacterial variety and richness were more significant in patients with cervical cancer. Among all the categories, *Lactobacillus* was the most prevalent. *Lactobacillus*, *Gardnerella*, and *Atopobium* decreased in response to HPV infection, but *Prevotella*, *Bacillus*, *Anaerococcus*, *Sneathia*, *Megasphaera*, *Streptococcus*, and *Anaerococcus* increased. The severity of CIN may have been influenced by a reduction in *G*. *vaginalis* and an increase in *Bacillus* and *Anaerococcus*. *Megasphaera* was significantly linked to HPV in the absence of CINs or malignant tumors. *Prevotella amnii* was the group’s most prevalent bacterium with low-grade squamous intraepithelial lesions (LSIL). At the family level, *Prevotella timonensis*, *Shuttleworthia*, and *Streptococcaceae* were linked to high-grade squamous lesions (HSIL) [70].

Mengying Wu et al. looked into whether the vaginal microbiota influences cervical squamous intraepithelial neoplasia development. They investigated sixty-nine women who came to the Obstetrics and Gynecology Hospital at Fudan University. Three groups of women were studied: those without intra-epithelial lesions or cancer, those with LSIL, and those with HSIL. The purpose of the study was to determine the makeup of vaginal bacteria in each group. In every group, *Lactobacillus* predominated. There were more streptococci and *prevotella* in the HSIL group. The development of cervical illness has been associated with the incidence of high-risk HPV infection. Those without intra-epithelial lesions or cancer had higher concentrations of the Pseudomonadales order, the Peptostreptococcaceae family, and other bacteria than those with squamous neoplasia. Comparing the LSIL and HSIL groups to the non-malignant group, Delftia enrichment is seen [71].

A 2021 research looked at the relationship between the emergence of SIL-related cervical cancer and the cervical microbiota of women who are of reproductive age. The research looked at 94 patients’ cervical mucous microbiota using 16 S rDNA. Alpha diversity was greater in severe cervical pathology, with less *Lactobacillus* and more anaerobes. Beta diversity varied greatly. *Sneathia* of HSIL and *Porphyromonas*, *Prevotella*, and *Campylobacter* of cervical cancer were marker genera. *Prevotella* had opposing functional pathway correlations with *Lactobacillus* [72].

The composition of vaginal bacteria in Mexican women with precancerous SIL was investigated by Nieves-Ramírez et al. The species richness rose, and the presence of SIL brought about compositional changes. The microbiota of HPV-positive women was altered even in the absence of SIL. According to research that used multivariate association with linear models (MaAsLin), HPV infection was associated with two operational taxonomic units (OTUs) reduction in *L*. *iner* and an increase in *Brachybacterium conglomeratum* and *Brevibacterium aureum* [73].

In five groups—normal, healthy, HR-HPV infections, LSIL, HSIL, and cervical cancer—Wei et al. looked at 59 Chinese women. Following HR-HPV infection and cervical lesions, there was a drop in *Lactobacillus* and an increase in the diversity of vaginal microbiota species. *Actinobacteria*, however, were significantly more prevalent in the four disease groups compared to the control group. Cervical cancer may also arise as a result of *L*. *iners*. After analyzing the whole process, *Gardnerella*, *Atopobium*, and *Dialister* impact HR-HPV persistence and etiology of cervical cancer [74].

Li et al.‘s 2023 study investigated cervical cancer, female vaginal microbiota, and immunological variables. *Gardnerella*, *Prevotella*, and *Lactobacillus* are the main bacteria in the vaginal flora. *Prevotella*, *Ralstonia*, *Gardnerella*, and *Sneathia* are more common in the group with cervical cancer than in the HPV-negative group. CIN with HPV positivity has a higher prevalence of *Gardnerella*, *Prevotella*, and *Sneathia* than non-CIN. The HPV-negative group has a preference for *Atopobium* and *Lactobacillus* [75]. Table 2 compiles the studies conducted on vaginal microbiota in cervical cancer patients.

**Table 2:** Vaginal microbiota in cervical cancer patients

Study	Number of participants and HPV groups	Microbiota specimen	Microbiota evaluation	Microbial change
Chen et al., 2020 [70]	229 patients and 51 LSIL, 23 HSIL, 9 ICC, 68 normal	lateral and posterior vaginal fornix	16 S rRNA sequencing	↑ *Prevotella*, *Bacillus*, *Anaerococcus*, *Sneathia*, *Megasphaera* and *Streptococcus* ↓ *Lactobacillus*, *Gardnerella*, and *Atopobium*
Mengying Wu et al., 2020 [71]	69 patients and 22 LSIL, 16 HSIL, 31 normal	Posterior vaginal fornix	16 S rRNA sequencing	↑ *Prevotella*, *Streptococcus* and *Delftia*
Sikao Wu et al., 2021 [72]	94 patients and 12 HPV+, 10 LSIL, 31 HSIL, 13 ICC, 28 normal	Cervix mouth	16 S rRNA sequencing	↑ *Sneathia*, *Porphyromonas*, *Prevotella*, and *Campylobacter* ↓ *Lactobacillus*
Nieves-Ramírez et al., 2021 [73]	228 patients and 121 SIL, 107 normal	Cervical swabs	16 S rRNA sequencing	↑ *Brachybacterium conglomeratum* and *Brevibacterium aureum* ↓ *L*. *iner*
Wei et al., 2022 [74]	59 patients and 13 HPV+, 5 LSIL, 10 HSIL, 11 CC, 10 normal	Cervical samples	16 S rRNA sequencing	↑ Actinobacteria, *Gardnerella*, *Atopobium* and *Dialister* ↓ *Lactobacillus*
Li et al., 2023 [75]	320 patients and 80 HPV + CIN, 80 HPV + non-CIN, 80 HPV-, 80 CC	Posterior vaginal fornix	16 S rDNA Sequencing	↑ *Prevotella*, *Ralstonia*, *Gardnerella*, and *Sneathia* ↓ *Lactobacillus* and *Atopobium*

Abbreviations: LSIL: low-grade squamous intraepithelial lesions, HSIL: high-grade squamous lesions, ICC: Invasive cervical cancer, SIL: squamous intraepithelial lesions, CC: cervical cancer.

### Ovarian cancer

The development of abnormal cell proliferation inside the ovaries characterises ovarian cancer. Cellular proliferation occurs rapidly, leading to the infiltration and subsequent degradation of normal bodily tissues. The female reproductive system comprises a pair of ovaries situated bilaterally next to the uterus [76]. Ovarian cancer is one of the leading causes of death for women with gynecological cancers. It also comes in at number five among cancers diagnosed in females [77]. In the year 2020, the incidence of ovarian cancer was estimated to be about 21,750 cases, accounting for around 1.2% of the total number of cancer cases. The anticipated fatality rate linked to the occurrence is 13,940. The projected relative survival rate over five years is estimated to be 48.6%. The study determined that the average incidence rate per 100,000 people, after adjusting for age using the 2000 US standard population, was 11.1 throughout the period spanning from 2012 to 2016. The demographic group exhibiting the highest incidence rate is people who identify as non-Hispanic white, with a rate of 11.6 per 100,000. Subsequently, American Indians and Alaska Natives have a rate of 10.3 per 100,000, followed by Hispanics with a rate of 10.1 per 100,000. Non-Hispanic blacks and Asian and Pacific Islanders also show varying rates [78].

The etiology of ovarian cancer is not fully comprehended, despite the presence of established risk factors including family history, age, inflammation, reproductive variables, benign gynecologic diseases, gynecologic surgery, and genetic susceptibility linked to mutations in the BRCA1 and BRCA2 genes [79, 80]. According to recent studies, the pathogenesis of ovarian cancer is significantly influenced by the vaginal microbiota.

Zhou et al. investigated the diversity and composition of the microbiota in normal distal fallopian tube tissues (25 samples) and ovarian cancer tissues (25 samples) using high-throughput sequencing techniques that targeted the 16 S rRNA gene. Compared to the normal distal fallopian tube tissues, the high-throughput sequencing study revealed a substantial decrease in diversity and richness indices within the ovarian cancer tissues. Furthermore, there was a notable increase in the relative abundance of two phyla in ovarian cancer cases: *Firmicutes* and *Proteobacteria*. The research cited above suggests that changes in the makeup of microbes may have a role in the development of ovarian cancer [81].

Two groups of women, ages 18–87, from the Czech Republic, Germany, Italy, Norway, and the United Kingdom were involved in the Nené et al. study. Patients with epithelial ovarian cancer and healthy, benign gynecological controls comprised the first group. The second group included wild-type BRCA1 and BRCA2 controls (healthy individuals with benign gynecological diseases) and women with BRCA1 mutations but no ovarian cancer. Every person’s cervicovaginal samples were sequenced for 16 S rRNA. It was calculated what percentage of lactobacilli species in the cervicovaginal microbiota offer a protective low vaginal pH. At the same time, community-type O samples had fewer than 50% lactobacilli, and community-type L samples contained at least 50%. In both groups, there was a stronger correlation seen in younger persons between the community type O microbiota and the status of ovarian cancer or BRCA1 mutation. Age-matched controls were less likely to have a community-type O microbiota than those with ovarian cancer and BRCA1 mutation carriers under 50. This risk would increase if more first-degree relatives developed cancer. Ovarian cancer risk factors were significantly correlated with the community-type O cervicovaginal microbiota, including age and BRCA1 germline mutations. According to the research, restoring a community-type L microbiota may help prevent ovarian cancer [82].

The retrospective investigation by Jacobson et al. found that ovarian cancer patients had a lower prevalence of vaginal communities dominated by *Lactobacillus*. This discovery was made in light of previously collected data on cancer-free women of comparable age [83].

Huang et al. examined 30 patients in 2022 to compare the intratumor microbiomes of epithelial benign ovarian tumors (EBOTs) with epithelial ovarian cancer (EOC). For this, high-throughput sequencing was used. Additionally, according to the study, Propionibacterium acnes may hasten the evolution of EOC. Compared to EBOT tissues, EOC tissues exhibit a more varied and abundant intratumor microbiome, according to high-throughput sequencing. EOC tissues had a higher abundance of *Actinomycetales*, *Acinetobacter*, *Streptococcus*, *Ochrobacterium*, and *Pseudomonas*. The study discovered that the *P*. *acnes* strain had a significant impact on the development of EOC [84].

In the comprehensive study conducted by Yu et al., intraoperative swabs were procured from the fallopian tube and other surgical sites to serve as controls. The research study included a total of 187 participants, consisting of 81 individuals diagnosed with ovarian cancer and 106 individuals without cancer. 1001 swabs were collected and submitted to 16 S rRNA gene PCR and sequencing to investigate the microbial composition. The study discovered a total of 84 bacterial species that have the potential to constitute the microbiota of the fallopian tube. A notable alteration was seen in the microbiome composition of individuals diagnosed with ovarian cancer in comparison to those without the disease. Certain bacterial strains, *Streptococcus parasanguinis*, and Neisseriaceae, were seen to be more prevalent in samples collected from the fallopian tubes and ovarian surfaces of individuals diagnosed with ovarian cancer. In contrast, several bacterial species, such as *Ruminiclostridium*, *Dialister invisus*, and *Bacteroides dorei*, were found solely in individuals diagnosed with ovarian cancer, although with a very low abundance [85]. Table 3 summarises the studies conducted on vaginal microbiota in ovarian cancer patients.

**Table 3:** Vaginal microbiota in ovarian cancer patients

Study	Number of participants	Microbiota specimen	Microbiota evaluation	Microbial change
Zhou et al., 2019 [81]	50	Ovarian cancer tissues	16 S rRNA sequencing and qPCR	↑ *Proteobacteria* and *Firmicutes*
Nené et al., 2019 [82]	580	Cervicovaginal smear samples	16 S rRNA sequencing and qPCR	↓ *Lactobacillus*
Jacobson et al., 2021 [83]	45	Vaginal samples	16 S rRNA sequencing	↓ *Lactobacillus*
Huang et al., 2022 [84]	30	EBOT and EOC tissue	16 S rDNA Sequencing	↑ *P. acnes*
Yu et al., 2023 [85]	187	Cervix, fallopian tubes, ovarian surfaces, and paracolic gutter samples	16 S rRNA sequencing and qPCR	↑ *S*. *parasanguinis*, *Neisseriaceae*, *Ruminiclostridium*, *D*. *invisus*, and *B*. *dorei*

### Endometrial cancer

Endometrial cancer is a malignancy that originates from the endometrium, which is the epithelial lining of the uterus or womb. The phenomenon under consideration is the outcome of an atypical proliferation of cells with the capacity to infiltrate or disseminate to distant anatomical sites [86, 87]. Endometrial cancer is a prevalent malignancy that affects a significant population of women worldwide [88]. In 2018, it was anticipated that this disease would cause 63,230 new instances of endometrial cancer and 11,350 deaths. According to this, endometrial cancer ranks as the fifth most common cause of cancer-related death in the US and the fourth most common cancer among women [89]. In terms of endometrial cancer incidence, white women exhibited the highest rates across various ethnic groups, with a recorded incidence of 24.8 cases per 100,000 individuals. Conversely, the death rate for black women was found to be twice as high as that of white women, with rates of 7.3 cases per 100,000 individuals compared to 3.9 cases per 100,000 individuals, respectively [90].

Endometrial cancer can be caused by several things, such as environmental factors, genetic predisposition, hormonal imbalances (especially involving estrogen and progesterone), heavy periods, being overweight, or old age. Recent research indicates that the vaginal microbiome has the potential to influence the development of endometrial cancer via direct or indirect mechanisms. This effect could happen by interacting with vulnerable endometrial tissue or by making different metabolites and inflammatory factors [52, 91].

In research conducted in 2019, Walsh et al. identified *Porphyromonas somerae* as the predominant microbial marker associated with endometrial cancer. Targeted qPCR was used to validate this finding, suggesting that it may be useful in identifying endometrial cancer in high-risk, asymptomatic women. It is essential to do more studies to investigate *P*. *somerae* and its related network’s possible role in the development of endometrial cancer, given the pathogenic qualities shown in the context of tissue infections and ulcers [92].

The study performed in 2022 by Barczyński et al. included a cohort of 48 female participants who presented with various medical conditions, including endometrial cancer, endometrial atypical hyperplasia, and benign gynecological problems. The research used real-time PCR to determine the bacterial species present in the samples. About specific species, it was shown that the incidence of *Mobiluncus curtisii* and *Fusobacterium nucleatum* was significantly elevated in vaginal samples. In contrast, the occurrence of *Gardnerella vaginalis* and *Fannyhessea vaginae* was mostly seen in samples obtained from the endocervical canal. It is of significance to observe that patients who have been diagnosed with endometrial cancer have a greater prevalence of vaginal microbiota as compared to their endocervical canal. However, the female participants in the control group had comparable levels of isolated bacteria in both the vaginal and endocervical canal swabs. The study showed a significant decrease in the prevalence of *Lactobacillus* spp. and *Bifidobacterium* spp. in cancer patients compared to the control group. Moreover, a notable disparity was seen in the number of microorganisms obtained from endocervical canal swabs between women belonging to the control group and those diagnosed with endometrial cancer [93].

Semertzidou et al. performed a comprehensive investigation of many anatomical locations within the reproductive system, including the vagina, cervix, endometrium, fallopian tubes, and ovaries, in a cohort consisting of 61 individuals. A microbial continuity was seen in the vaginal tract of the majority of women without cancer, as shown by the consistent presence of bacterial species often found in this region. A correlation has been shown between endometrial cancer and a reduction in the presence of bacteria in both the cervicovaginal and rectal regions. Subsequently, there was a decline in the relative prevalence of *Lactobacillus* species, namely *L*. *crispatus*. Furthermore, an increase in the variety of bacteria was seen in conjunction with the expansion of several bacterial taxa, such as *Porphyromonas*, *Prevotella*, *Peptoniphilus*, and *Anaerococcus*, in both the lower genital tract and endometrium of persons who have been diagnosed with endometrial cancer [94].

The study done by Barczyński et al. included a cohort of 96 participants who had surgical interventions for a range of illnesses, such as benign uterine disorders, precancerous endometrial lesions, and endometrial cancer. The researchers used real-time PCR analysis to detect and measure the abundance of 19 commonly occurring bacteria in samples collected from the vaginal fornix and endocervical canal. Microbial presence was observed in 88.5% of vaginal samples and 83.3% of cervical samples. The prevalence of *L*. *iners* was higher among those with benign diseases, but *Dialister pneumosintes* and *Mobiluncus curtisii* were found to be more prevalent among cancer patients. The prevalence of these two bacteria was notably higher in vaginal samples obtained from individuals with endometrial cancer, suggesting a possible role in the initiation or promotion of carcinogenesis. Nevertheless, the precise process remains elusive, requiring further investigation [95]. Table 4 summarises the studies conducted on vaginal microbiota in endometrial cancer patients.

**Table 4:** Vaginal microbiota in endometrial cancer patients

Study	Number of participants	Microbiota specimens	Microbiota evaluation	Microbial change
Walsh et al., 2019 [92]	151	Vaginal swabs, cervical swabs, uterine, fallopian, and ovarian samples	16 S rRNA sequencing and qPCR	↑ *P*. *somerae*
Barczyński et al., 2022 [93]	48	Vaginal and endocervical swabs	Real-Time PCR	↓ *Lactobacillus* and *Bifidobacterium*
Semertzidou et al., 2022 [94]	61	Vagina, cervix, endometrium, fallopian tubes, and ovaries swabs	16 S rRNA sequencing	↑ *Porphyromonas*, *Prevotella*, *Peptoniphilus*, and *Anaerococcus* ↓ *L*. *crispatus*
Barczyński et al., 2023 [95]	96	Vaginal and cervical swabs	Real-Time PCR	↑ *D*. *pneumosintes* and *M*. *curtisii* ↓ *L*. *iner*

### Vaginal cancer

Vaginal cancer is precisely characterised as a medical condition that lacks any indication of cervical or vulvar cancer or a documented history of either during the preceding five-year period. The majority of vaginal lesions (about 80–90%) are derived from cervical or vulvar lesions, as well as other nearby locations such as the endometrium, bladder, rectosigmoid, or ovary [96, 97]. Vaginal cancer is a very uncommon kind of malignancy, accounting for around 1–2% of all gynecologic cancers [98]. According to a survey provided by the American Cancer Society, it is projected that around 8,180 new instances of vaginal cancer will be detected in the year 2021. The projected fatality rate is estimated to be around 1530 individuals among the total number of diagnosed cases [99].

Squamous cell carcinoma is the predominant histological subtype of primary vaginal cancer, comprising about 90% of the total incidence. Additional, less prevalent variants include adenocarcinoma, clear cell adenocarcinoma, melanoma, sarcoma, and lymphoma affecting the vaginal region [100]. Vaginal cancer is correlated with several risk factors, such as infection with human papillomavirus (HPV) and herpes simplex virus (HSV), exposure to diethylstilbestrol, chronic irritation, prior radiation therapy, and an imbalance in the homeostatic equilibrium of the vaginal microbiota [52, 101].

A correlation has been shown between the composition of the vaginal microbiota and the occurrence of vaginal intraepithelial neoplasia (VAIN). The progression of VAIN and the development of vaginal cancer have been associated with changes in the composition of the vaginal microbiota, including an increased presence of *Atopobium*, *Gardnerella*, *Enterococcus*, *Clostridium*, and *Allobaculum*. Additionally, an elevated viral load of HPV-16, 52, and 58 has been identified as a contributing factor [9].

### Vulvar cancer

Vulvar cancer is considered a relatively uncommon malignancy within the field of gynecology, accounting for around 4% of all malignancies affecting the female genital system [102]. Based on the figures presented by the International Agency for Research on Cancer (IARC), the yearly incidence of vulvar cancer exceeds 45,000 cases, with about 50.1% of these occurrences seen in countries with high-income economies [103]. This particular neoplasia is accountable for an estimated annual mortality rate of 17,000 individuals, primarily concentrated in high-income nations (40.8% of cases) [102].

**Fig. 2:**
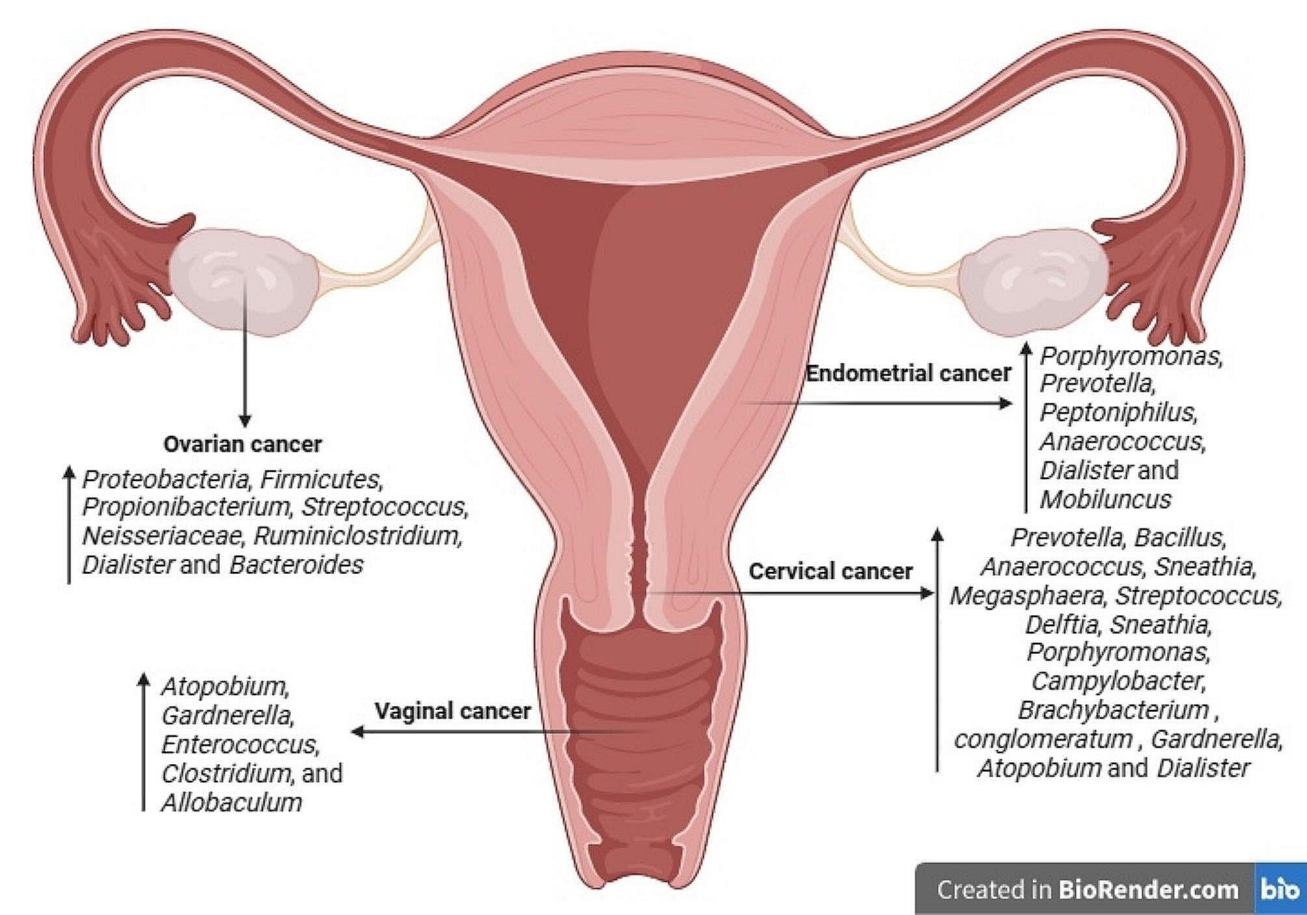
Alteration of the female vaginal microbiota throughout gynecological cancers

Squamous cell carcinoma accounts for around 90% of vulvar malignancies. Less common histologic subtypes include basal cell carcinoma, verrucous carcinoma, Bartholin’s gland adenocarcinoma, extramammary Paget’s disease, and vulvar melanoma [102]. Various risk factors have been identified about the development of vulvar cancer. These include advancing age, infection with HPV, tobacco smoking, inflammatory vulvar diseases, previous pelvic radiation, and compromised immune function [104]. The presence of mucosal HPVs has been associated with the development of vulvar cancer in young women who have previously had genital warts, cervical dysplasia, and immunosuppression. The majority of vulvar cancer patients who test positive for HPV carry the HPV-16 strain, accounting for around 80–90% of cases. The remainder of individuals exhibit HPV18 or HPV33 [105]. Figure 2 illustrates the Alteration of the female vaginal microbiota throughout gynecological cancers.

## Conclusions

Epidemiological research has shown a significant correlation between the makeup of the vaginal microbiota and the incidence of gynecological cancers. However, our understanding of the host’s defensive mechanisms against these microorganisms remains limited. Additional investigation is required to clarify the functional implications of these microbial communities on the vaginal microenvironment, particularly concerning tumor formation. Future investigations using extensive clinical datasets, in conjunction with in vitro models encompassing both human and animal participants, will assume a pivotal role in understanding the exact impact of these microorganisms on the genesis and advancement of gynecological malignancies. This will provide a more comprehensive comprehension of these bacteria’ functions in the beginning and progression of gynecological malignancies. In summary, a deeper understanding of the intricate interplay between the host and microorganisms in the vaginal area holds promise for uncovering novel strategies in the realm of cancer prevention and therapy. Consequently, this would enhance women’s overall welfare and physical state.

## Data Availability

No datasets were generated or analysed during the current study.
